# Keeping your armour intact: How HIV-1 evades detection by the innate immune system

**DOI:** 10.1002/bies.201400019

**Published:** 2014-04-29

**Authors:** Jonathan Maelfait, Elena Seiradake, Jan Rehwinkel

**Affiliations:** 1 Medical Research Council Human Immunology Unit, Medical Research Council Weatherall Institute of Molecular Medicine, Radcliffe Department of Medicine, University of OxfordOxford, UK; 2) Division of Structural Biology, Wellcome Trust Centre for Human Genetics, University of OxfordOxford, UK

**Keywords:** cGAS, CypA, cytosolic DNA sensor, dendritic cells, HIV-1, HIV-1 capsid, innate antiviral immunity

## Abstract

HIV-1 infects dendritic cells (DCs) without triggering an effective innate antiviral immune response. As a consequence, the induction of adaptive immune responses controlling virus spread is limited. In a recent issue of *Immunity*, Lahaye and colleagues show that intricate interactions of HIV capsid with the cellular cofactor cyclophilin A (CypA) control infection and innate immune activation in DCs. Manipulation of HIV-1 capsid to increase its affinity for CypA results in reduced virus infectivity and facilitates access of the cytosolic DNA sensor cGAS to reverse transcribed DNA. This in turn induces a strong host response. Here, we discuss these findings in the context of recent developments in innate immunity and consider the implications for disease control and vaccine design.

## Introduction

A striking quality of HIV-1 is its ability to enter dendritic cells (DCs) – a specialised subset of antigen presenting cells – without triggering an innate immune response, despite the fact that these cells readily sense infection with a wide range of other pathogens, including HIV-2. Indeed, productive infection of DCs with HIV-1 is inefficient, and this has been attributed to SAMHD1, a host restriction factor that inhibits viral replication at the level of reverse transcription 1; and to the redirection of incoming virus into a degradative, non-productive route of infection 2–5. When the block on cDNA synthesis is alleviated by depletion of SAMHD1, a strong antiviral immune response is induced in DCs 6. Recent evidence shows that HIV-1 capsid (CA) and its interaction with cellular proteins such as cyclophilin A (CypA) and CPSF6 further define whether virus is sensed or not 7,8. CA mutations that modulate binding to these cofactors affect the integrity of the CA core and perturb nuclear import. As a consequence, reverse transcribed cDNA gains access to the cytosol, where it activates the cytosolic DNA receptor cGAS that induces a potent antiviral immune response. This review highlights recent progress in innate sensing of HIV-1 and the implications that these findings have for our understanding of HIV-1 pathogenesis and control.

## Interactions between HIV-1 CA and host cofactors direct the early infection process

**Figure 1:**
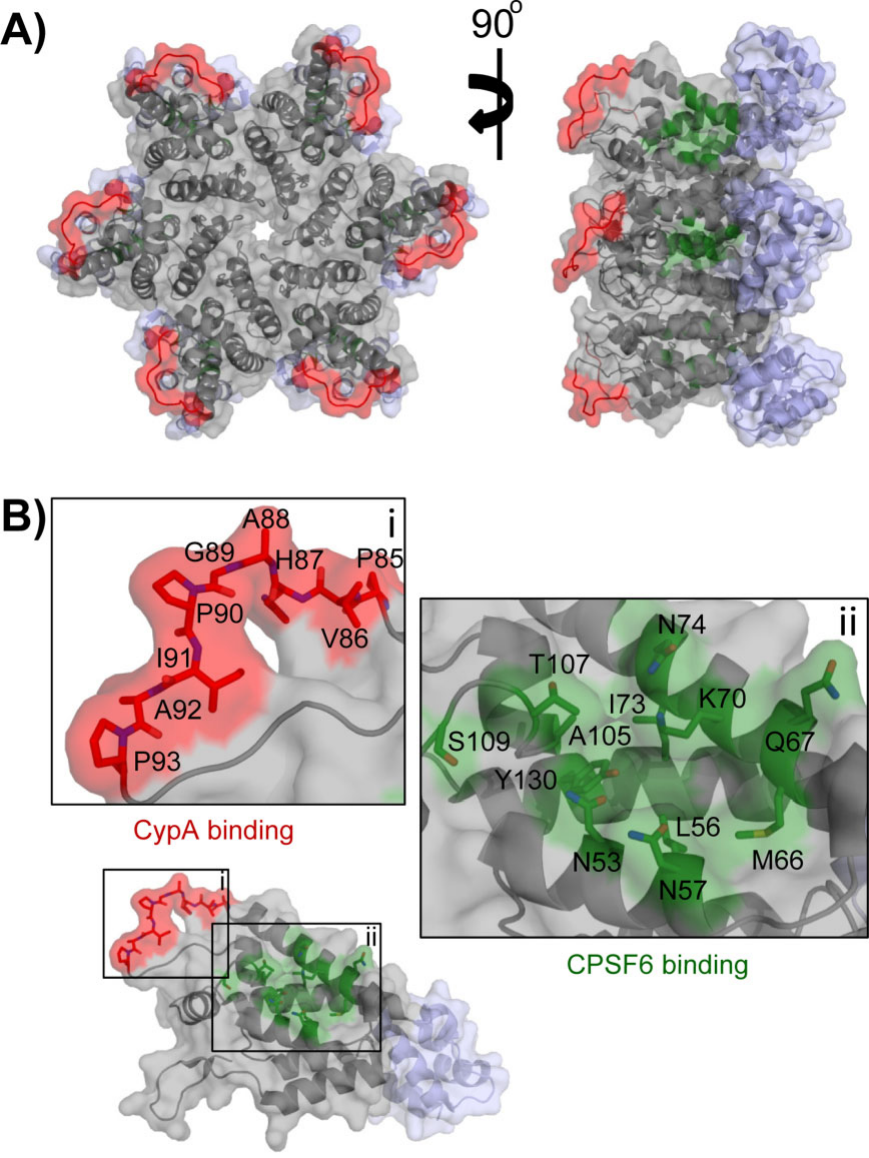
Structure of the HIV-1 capsid hexamer. A: A structural model of the HIV-1 capsid (CA) hexamer was generated based on the Protein Data Base (PDB) entries 1AK4 17 and 3H47 9. A backbone ribbon and surface view is shown; grey = N-terminal domain (residues 1-146), light blue = C-terminal domain (residues 147-219). Residues of the cyclophilin binding loop 17 and the CPSF6 binding site 84 are coloured red and green, respectively. B: View of the CA monomer with colours as in panel A. Residues forming the cyclophilin binding loop and the CPSF6 binding site are shown as stick models. Note that for the CPSF6 binding site we show only the side chain atoms as sticks; the backbone atoms are shown as ribbons.

Mature, cell-free HIV-1 particles have a membranous envelope that surrounds a characteristic conical core, which in turn contains the viral RNA genome. This core is made up of ∼1,500 viral CA proteins assembled into hexameric rings 9. Twelve CA pentamers located at the top and bottom edges of the lattice give the CA core flexibility to assume its geometry 10. HIV-1 CA not only maintains the structural integrity of the virus but is also involved in some steps of the infection cycle. CA is a determinant of the ability of HIV-1 to infect non-dividing cells 11,12. In particular, two protein interaction motifs have been defined on the outer surface of the mature HIV-1 CA lattice (Fig. 1A). These interfaces – along with the host cofactors they bind – assure timely uncoating, correct assembly of the reverse transcription complex (RTC), and its transport across the nuclear membrane 13.

One protein interaction interface of HIV-1 CA is the cyclophilin-binding loop, a protruding proline rich stretch of amino acids within the N-terminal domain (NTD) (Fig. 1B). This motif, encompassing residues 85–93, binds with high affinity to the cytosolic protein CypA, a peptidyl prolyl isomerase 14. The C-terminus of NUP358, a nuclear pore complex protein and HIV-1 host cofactor, also adopts a cyclophilin domain-like structure that binds to HIV-1 CA 15,16. CA residues G89 and P90 within the cyclophilin-binding loop are critically important for CypA and NUP358 binding, and directly interact with the catalytic pockets of these proteins 15,17. Knockdown of CypA or NUP358 – as well as mutation of G89 and P90 – disrupt nuclear import of the RTC and reduce virus replication 15,18,19. This demonstrates an important role of these protein-protein interactions during the early infection process. Both CypA and NUP358 isomerise the G89-P90 peptide bond from a cis to a trans conformation 20,21. Although the exact biological function of CA isomerisation is unknown, it is thought to destabilise CA cores in preparation for the uncoating process.

A second protein interaction interface lies within a crevice of the NTD of HIV-1 CA adjacent to the cyclophilin-binding loop and binds to the host cofactors CPSF6 and nuclear pore protein NUP153 (Fig. 1B). The N-terminus of CPSF6 can bind directly to CA 22,23, while the C-terminal arginine/serine-rich (RS) domain bridges CA to another cofactor, TNPO3, which mediates nuclear transport of the RTC 24–28. C-terminally truncated CPSF6, lacking the RS-domain, is unable to interact with TNPO3 and acts as a pre-integration restriction factor 22. CA core uncoating and reverse transcription occur in parallel and are mutually dependent processes 29,30. The short form of CPSF6 prevents uncoating and stabilises the CA core inside the cytosol and as such reverse transcription is blocked 26. The NUP153 interaction motif in CA overlaps with the CPSF6 binding site, suggesting that both proteins act in concert to mediate nuclear transport of the RTC 31–33. CA N74D mutant virus can escape restriction from truncated CPSF6, but shows impaired growth compared to wild-type virus 15,22,34. Protein structure analysis shows that N74 lies within the CPSF6-binding interface of HIV-1 CA (Fig. 1B) and is crucial for CPSF6 binding 35. The N74D mutation impairs the interactions with CPSF6/TNPO3 and probably also NUP153, thus targeting the RTC to suboptimal nuclear pore complexes.

Taken together, CA interactions with host proteins and the analysis of CA mutant viruses show that HIV-1 CA plays an important role during early steps of infection. Direct binding of cellular cofactors such as CypA, CPSF6, NUP358 and NUP153 directs capsid uncoating, reverse transcription and nuclear import. Furthermore – as we will discuss below – controlled capsid uncoating also shields the reverse transcribed DNA from detection by the innate immune system.

## Recent advances in cytosolic DNA sensing

**Figure 2:**
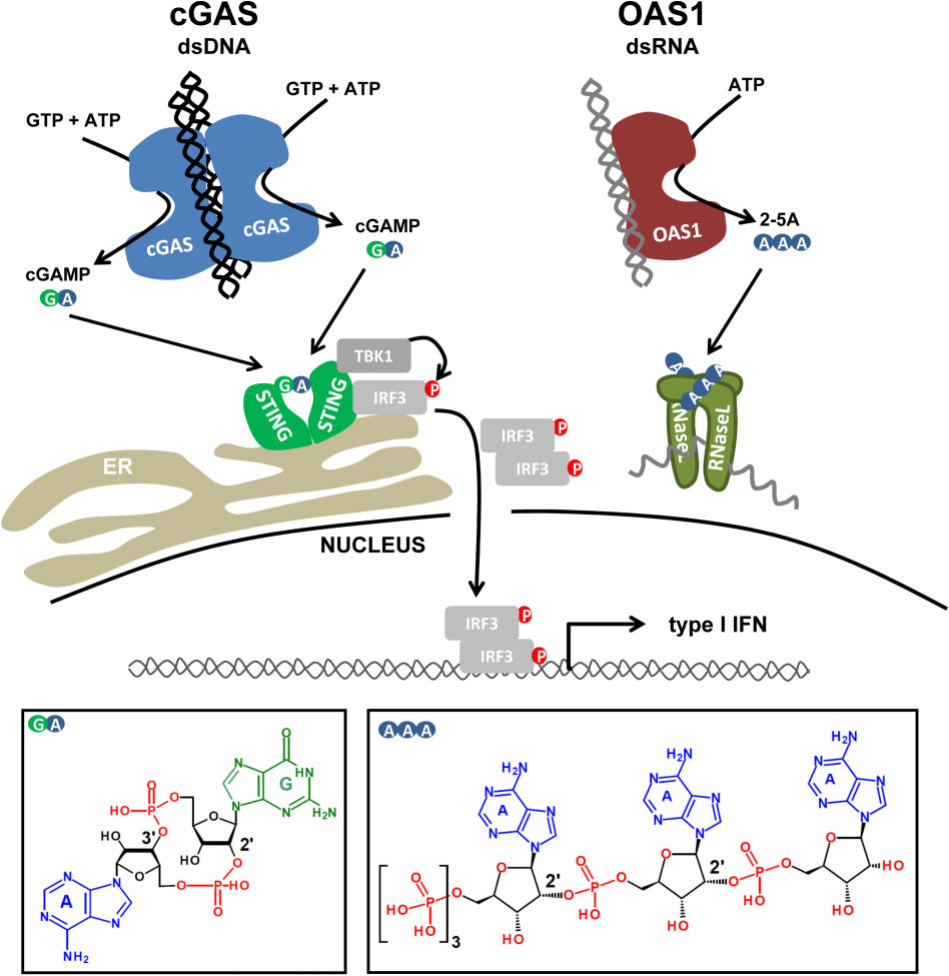
Comparison of cGAS and OAS1 signalling pathways. Left: binding of DNA to cGAS occurs in a 2:2 conformation and induces a structural rearrangement in the catalytic pocket of cGAS. As a result cGAMP [cyclic G(2′,5′)pA(3′,5′)p, chemical structure on bottom left] is synthesised from its substrates GTP and ATP. cGAMP then binds to dimeric STING located at the endoplasmic reticulum (ER) membrane. This allows docking of the kinase TBK1 and of the transcription factor IRF3, leading to IRF3 phosphorylation (P) by TBK1. IRF3 then forms a dimer and translocates to the nucleus, where it induces the transcription of type I IFN. Right: OAS1 is activated upon binding to dsRNA. This opens up the catalytic pocket, where a linear 2′,5′-linked oligoadenylate chain [ppp5'(A2'p5')2A or 2-5A, chemical structure on the bottom right depicts a 2-5A trimer] is formed. 2-5A induces dimerisation and activation of RNaseL, which cleaves cellular and viral RNA. Note the 2′,5′ phosphodiester bonds between GMP (G) and AMP (A) in cGAMP or between two AMP molecules in 2-5A. Chemical structures were drawn with Accelrys Draw.

The presence of DNA in the cytosol is detected by the innate immune system and triggers a protective host response. Multiple proteins have been identified as candidate cytosolic DNA sensors including DAI 36, IFI16 37, DDX41 38, RNA Pol III 39,40 and the DNA damage proteins MRE11 41, DNA-PK 42 and Ku70 43. The function of some of these receptors, however, appears to be species or cell type specific, and in vivo evidence for their function is often lacking. Downstream signalling involves the kinase TBK1, which phosphorylates and thereby activates the transcription factor IRF3 44,45. Upon translocation to the nucleus, IRF3 induces the expression of antiviral genes including those encoding type I interferons (IFN), a typical feature of antiviral immunity (Fig. 2).

### The discovery of the cGAS-STING signalling axis

The endoplasmic reticulum transmembrane protein STING is a mediator for cytosolic DNA sensing 46–49 (Fig. 2). STING lacks a high-affinity DNA binding domain 50 and hence may not be a direct DNA sensor. Interestingly, STING induces a host response after direct binding to the bacterial second messenger molecules cyclic-di-GMP (c-di-GMP) and c-di-AMP 51–54. In vivo evidence shows that this is an important defence mechanism against the cytosolic bacterium *Listeria monocytogenes* 54,55. The dual function of STING, acting both as receptor for bacterial cyclic di-nucleotides and also playing a central role in DNA sensing, remained poorly understood until ground-breaking work in the Chen lab discovered the enzyme cyclic GMP-AMP synthase (cGAS) as a sensor for cytosolic DNA 56. Instead of conveying downstream signalling by classical protein-protein interactions, cGAS catalyses the synthesis of a diffusible second messenger molecule called cyclic-GMP-AMP (cGAMP) upon allosteric activation by DNA 57. cGAMP is a high affinity ligand for STING and induces a change in its conformation allowing for the formation of a signalling platform that activates IRF3 via its kinase TBK1 58–61 (Fig. 2). cGAS knockout mice are highly susceptible to DNA viruses and primary cells isolated from these mice fail to respond to cytosolic DNA stimuli, highlighting the importance of this protein in immune defence against viruses 62,63.

### Direct binding of DNA to cGAS triggers cGAMP production

A combination of structural and biochemical studies provided detailed mechanistic insight into how cGAS catalyses the synthesis of cGAMP 64–69. cGAS adopts a bilobal structure comprising a nucleotidyltransferase fold and two DNA binding sites. Upon DNA binding, cGAS forms a complex in which two opposing cGAS proteins interact with two DNA molecules. Importantly, DNA binding induces a structural rearrangement in the catalytic pocket that enables the enzymatic activity of cGAS. Analysis of the complex between cGAS, DNA and its substrates ATP and GTP shows that the nucleotidyltransferase domain of cGAS forms a 2′-5′ phosphodiester linkage between the 2′-hydroxyl group of GTP and the 5′-position of ATP, before closing the dinucleotide ring with a 3′-5′ phosphodiester bond. The product of this two-step reaction is cGAMP. cGAS shows structural similarities with the antiviral protein OAS1, which upon binding to double stranded RNA catalyses the formation of 2′-5′ phosphodiester bonds between ATP molecules, resulting in the formation of 2′-5′-oligoadenylate (2-5A) 70,71. 2-5A binds and activates RNaseL, an endoribonuclease that non-specifically cleaves viral and cellular RNA molecules, thereby inhibiting replication of several RNA viruses 72 (Fig. 2).

These discoveries have changed our understanding of innate immunology. For example, retroviruses reverse transcribe their genomic RNA into double stranded cDNA, a process that takes place within the cytosol and had been predicted to trigger cytosolic DNA sensors. Indeed, IFI16 detects abortive reverse transcription products in resting CD4^+^ T cells, which initiates a pro-inflammatory form of programmed cell death called pyroptosis 73,74. Moreover, cGAS is involved in sensing of reverse transcribed DNA products and IFN induction during HIV-1 infection in cell lines 75,76. However, the role of cGAS in natural HIV-1 infection of primary target cells such as macrophages and DCs, which are not activated upon virus entry, and the evasion strategies employed by the virus, have not been investigated in detail.

## Evasion of innate immunity by HIV-1 capsid

### Degradation of SAMHD1 by Vpx allows innate detection of HIV-1 in DCs

**Figure 3:**
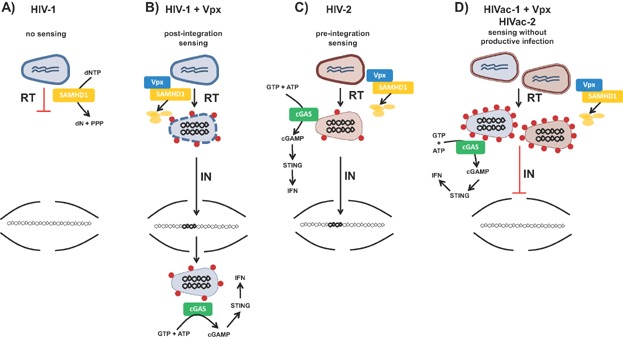
The role of CA and Vpx in sensing of HIV-1 and HIV-2 in DCs. A: HIV-1 infection of DCs does not result in innate sensing, because reverse transcription (RT) of the viral genome is blocked by the host restriction factor SAMHD1, which depletes the cellular dNTP pool. B: Administration of Vpx to DCs at the time of HIV-1 infection results in the degradation of SAMHD1. As a result, reverse transcription can proceed, leading to productive infection of DCs. Balanced interactions between CypA (red spheres) and HIV-1 CA prevent sensing of HIV-1 cDNA before integration (IN). Interaction of newly synthesised CA with CypA may allow remaining cytosolic cDNA to be detected by cGAS, leading to cGAMP production and type I IFN production. C: HIV-2 naturally expresses Vpx, enabling productive infection of DCs. Low-affinity binding of CypA to HIV-2 CA results in exposure of reverse transcription products to the cytosol, where they are detected by cGAS before integration of viral cDNA. D: Infection of DCs with affinity enhanced CA HIV-1 (HIVac-1) in combination with Vpx delivery or with HIVac-2 induces an innate antiviral immune response without integration of viral cDNA. Enhanced CypA binding to modified CA causes CA core destabilisation in the cytosol and detection of reverse transcribed DNA by cGAS. HIVac-1 and HIVac-2 fail to integrate into the genome probably due to disruption of nuclear import.

Infection of DCs with HIV-1 is enhanced by delivery of the macaque SIV accessory protein Vpx 77. Vpx targets the host restriction factor SAMHD1 for proteasomal degradation 78,79. SAMHD1 is a deoxyribonucleotide (dNTP) triphosphohydrolase, and depletes cellular dNTP levels by cleaving dNTPs into deoxynucleosides and inorganic triphosphates 80,81. This constitutes an antiviral defence mechanism by limiting the availability of dNTPs required for reverse transcription 82. Littman, Manel and colleagues were the first to show that co-infection of DCs with HIV-1 and virus like particles carrying Vpx not only overcomes virus restriction, but also induces an antiviral immune response 6. In this system, sensing of HIV-1 requires reverse transcription and integration, and is also dependent on the interaction of newly synthesised HIV-1 CA with CypA (Fig. 3A and B).

### The timing of innate sensing in DCs is determined by HIV CA

**Figure 4:**
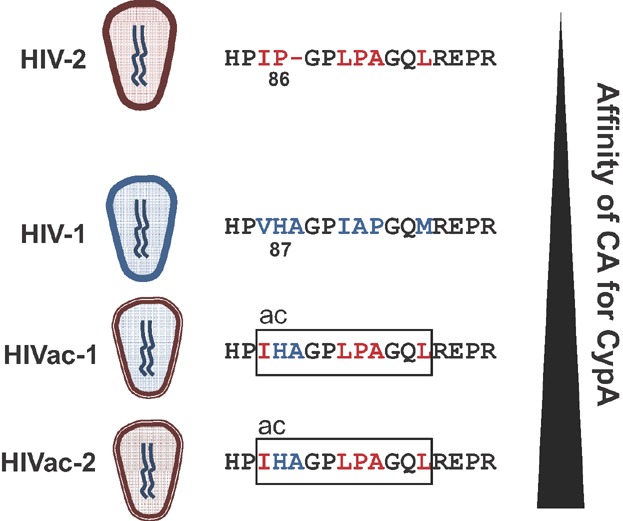
Comparison of the cyclophilin-binding loops of affinity enhanced CA viruses (HIVac-1 and HIVac-2) with wild-type viruses. Unique HIV-2 or HIV-1 amino acids are depicted in red or blue, respectively. HIV-2 CA has a low affinity for CypA and replacing Pro86 in the cyclophilin-binding loop with the HIV-1 sequence His87-Ala88 generates a chimeric CA with greatly enhanced CypA affinity. Grafting the modified HIVac-2 cyclophilin-binding loop (boxed sequence, ac) into HIV-1 CA generates HIVac-1, which has greater affinity for CypA than the wild-type virus.

HIV-2 naturally expresses Vpx and efficiently infects DCs. In contrast to HIV-1, activation of DCs by HIV-2 does not depend on integration of viral cDNA 6 (Fig. 3C). These data indicate that, although Vpx – and thus reverse transcription – is prerequisite for sensing of both HIV-1 and HIV-2, a different viral factor determines whether sensing occurs pre-integration or not. In a recent issue of *Immunity*, Manel and colleagues postulated that an inherent difference in CA between the two viruses might explain this difference 7. Indeed, HIV-2 CA has reduced affinity for CypA compared to HIV-1 CA 83,84. To study the impact of CA on innate immune activation, the authors generated a mutant HIV-2 in which the cyclophilin-binding loop of the CA protein partially resembles that of HIV-1, thereby greatly enhancing its affinity for CypA (HIV-2 affinity enhanced capsid; HIVac-2, Fig. 4). HIVac-2 does not productively infect DCs. Importantly; the mutant virus is still sensed by DCs, and it triggers DC activation and IFN induction (Fig. 3D). Reverse transcription of viral genomic RNA is crucial for innate detection of HIVac-2, whereas integration is dispensable.

To address whether CA and its affinity for CypA also defines sensing of HIV-1, Lahaye and colleagues substituted the cyclophilin-binding loop of HIV-1 with the sequence of HIVac-2. This CA mutated HIV-1 (HIVac-1) has a slightly increased affinity for CypA compared with wild-type HIV-1 CA (Fig. 4). Similar to HIVac-2, HIVac-1 is strongly attenuated in its ability to productively infect DCs. Nevertheless, it activates DCs and induces an antiviral immune response with greater efficiency than wild-type HIV-1. The detection of HIVac-1 is dependent on the addition of exogenous Vpx, indicating a requirement for reverse transcription. However, in contrast to wild-type HIV-1, it is independent of integration and newly synthesised CA, thus mimicking the situation seen with HIV-2 (Fig. 3C and D).

Mutations in the cyclophilin-binding loop of CA not only disturb interactions with CypA, but probably also with NUP358. Disrupted interactions between CA and CypA/NUP358 affect CA stability and inhibit nuclear import of the RTC. Indeed, during HIVac-2 infection, reverse transcription remains unaffected, while the presence of 2LTR circles – a marker for nuclear entry – and the copies of integrated provirus are both reduced, indicating that nuclear import is impaired. Taken together, these results show that balanced interactions between viral CA and host cofactors determine infectivity, and when and how HIV is sensed in DCs.

### cGAS detects reverse transcription products in DCs

The authors next asked which innate signalling pathways detect HIV in DCs. Given the requirement for Vpx, which enables efficient reverse transcription, Lahaye et al. tested the role of different candidate cytosolic DNA sensors using shRNA knockdown strategies. These experiments revealed that cGAS is essential for DC activation after HIV-2, HIVac-2 and HIV-1 infection in the presence of Vpx. This result is consistent with earlier work demonstrating a requirement for STING in sensing of HIV-1 in other cell types 85,86. These observations raise a number of interesting questions for future studies: Why is integration required for sensing of HIV-1 in DCs? Why can cGAS sense HIV-2 (and HIVac-1) reverse transcribed DNA before integration? In the case of wild-type HIV-1, reverse transcription and nuclear import may be tightly linked processes. A CA core supported by optimal CA-CypA/NUP358 interactions may shield the process of reverse transcription from cGAS, starting from entry of the virus into the cytosol up until nuclear import. Indeed, intact CA cores can be detected at the nuclear pores 87. Such synchronisation of reverse transcription, CA uncoating and nuclear import may not occur as efficiently in the case of HIV-2, HIVac-1 and HIVac-2, allowing cGAS to gain access to the RTC before integration. Lahaye et al. further raise the possibility that during infection with wild-type HIV-1, interactions between newly synthesised CA and CypA result in unmasking of remnant cDNA in the cytosol after integration, which only then triggers cGAS (Fig. 3B). It will also be interesting to determine whether the actual DNA that triggers cGAS corresponds to fully reverse-transcribed cDNA, intermediates of reverse transcription or to aberrant, non-productive RT products. Indeed, the latter scenario seems to play an important role during infection with another retrovirus, human T cell leukaemia virus type 1 88. It should be noted that the above experiments were performed using VSV-G pseudotyped replication defective lentivectors. During natural infection, the HIV envelope proteins gp120/gp41 interact with the cell surface receptors CD4 and CCR5 and mediate entry through fusion rather than endocytosis as is the case for VSV-G pseudotyped virions 89,90. Although VSV-G pseudotyping does not seem to interfere with post-integration sensing of wild-type HIV-1 6, it is currently unclear whether pre-integration sensing of reverse transcription products is affected by the route of entry.

### CA plays an important role in HIV-1 sensing in macrophages

In addition to DCs, other myeloid cells such as macrophages are also important target cells for HIV-1 and play a role in virus induced immunopathology and progression to AIDS 91. Similar to DCs, HIV-1 infection of macrophages occurs without inducing antiviral immunity 92. Recent findings by Towers and colleagues show that the ability of HIV-1 CA to interact with host cofactors is a strategy to prevent detection by the innate immune system 8. N74D and P90A CA mutant viruses, which are unable to recruit CPSF6/NUP153 and CypA/NUP358, respectively, do not replicate in macrophages as a result of type I IFN mediated restriction. This study also pinpoints reverse transcribed cDNA as the trigger for the host antiviral response. The mechanism by which IFN blocks replication was not tested; however, the recently identified IFN inducible HIV-1 restriction factor Mx2 might contribute to the restriction of CA mutant viruses in macrophages 93–95. This IFN feedback mechanism may also in part explain the reduced infectivity of CA mutated virus in DCs.

In summary, these findings show that HIV-1 has surrounded its nucleic acids with a CA core that allows the virus to evade detection by the innate immune system in DCs and macrophages. Sensing of HIV-2 in DCs is attributable to Vpx, which allows reverse transcription to proceed efficiently, and a suboptimal CA structure, which permits the cytosolic DNA sensor cGAS to gain access to reverse transcribed cDNA and to trigger a host response.

## Conclusions and prospects

Superior DC activation by HIV-2 compared to HIV-1 might be an explanation for the decreased pathogenicity and better immune control observed in HIV-2 infection 96,97. The reverse question can also be asked: whether the more severe pathology of HIV-1 is attributable to the absence of Vpx and to evolutionary fine-tuning of CA-cofactor interactions that avoid innate immune detection. Clues may come from studying natural HIV-1 CA variants and their capacity to stimulate host responses.

Lahaye et al. also show that DCs infected with HIVac stimulate CD4^+^ and CD8^+^ T cell activation in the absence of productive infection. This has interesting implications for vaccine design. Attenuated virus such as HIVac-1 might prove advantageous for several reasons. First, antigen and adjuvant (reverse transcribed DNA) are co-delivered to antigen presenting cells. Second, activation of DCs through the cGAS pathway stimulates virus specific T cells. Finally, HIVac carries the same antigenic epitopes as wild-type virus. Efficient co-delivery of Vpx and HIVac-1 to DCs might be a limiting factor. Packaging of Vpx into HIV-1 virions or incorporation of Vpx into the HIV-1 genome allows for efficient infection of DCs and should thus be applicable to the HIVac-1 system 98.

However, enhancing innate immune responses may not always be beneficial. Sensing of reverse transcripts by IFI16 in quiescent CD4^+^ T cells induces cell death, which is thought to contribute to CD4^+^ T cell loss and progression to AIDS 73,74. TLR-mediated cytokine secretion by plasmacytoid DCs attracts CD4^+^ T target cells to the site of infection 99. Moreover, innate immune driven chronic inflammation is predictive of negative disease outcome, and contributes to HIV-1 associated immunopathogenesis 100. Treatment strategies that boost the immune response should therefore be approached with caution, and further research should determine whether improving HIV-1 sensing in DCs is a valid approach to HIV-1 control.

## Abbreviations

CA: capsid protein
CypA: cyclophilin A
DC: dendritic cell
HIV: human immunodeficiency virus
HIVac: HIV affinity enhanced capsid
IFN: interferon
RTC: reverse transcription complex
